# Lightweight similarity checking for English literatures in mobile edge computing

**DOI:** 10.1186/s13677-022-00384-5

**Published:** 2023-01-05

**Authors:** Xiaomei Liu, Ailing Gao, Chengxiang Chen, Mohammad Mahdi Moghimi

**Affiliations:** 1 Shandong Provincial University Laboratory for Protected Horticulture, Weifang University of Science and Technology, Weifang, China; 2 Fujian Polytechnic Normal University, Fuzhou, China; 3 Department of Electrical Engineering, Yazd Branch, Islamic Azad University, Tehran, Iran

**Keywords:** Mobile edge computing, Literature similarity checking, Hash index, Lightweight

## Abstract

With the advent of information age, mobile devices have become one of the major convenient equipment that aids people’s daily office activities such as academic research, one of whose major tasks is to check the repetition rate or similarity among different English literatures. Traditional literature similarity checking solutions in cloud paradigm often call for intensive computational cost and long waiting time. To tackle this issue, in this paper, we modify the traditional literature similarity checking solution in cloud paradigm to make it suitable for the light-weight mobile edge environment. Furthermore, we put forward a lightweight similarity checking approach $\mathrm {SC_{MEC}}$ for English literatures in mobile edge computing environment. To validate the advantages of $\mathrm {SC_{MEC}}$, we have designed massive experiments on a dataset. The reported experimental results show that $\mathrm {SC_{MEC}}$ can deliver a satisfactory similarity checking result of literatures compared to other existing approaches.

## Introduction

Benefiting from the continuous progress of mobile computing technology, various mobile devices (e.g., smart phones, PDA, etc.) have engaged into the daily life of people and are more and more crucial in people’s entertainment, job, shopping and so on [1–3]. Such a mobile life manner brings many conveniences to people since it minimizes the negative influences brought by physical space of different people [4–7]. Specifically, with the advent of COVID-19 pandemic, people’s activity ranges are further limited. In this situation, mobile devices have gradually become one of the major ways to aid people’s various activities including academic research [8–10]. Today, more and more people are apt to take mobile devices as their major academic research tools and hence generate a series of mobile devices-enabled academic research tools such as online academic meeting, multi-party academic collaborations, etc. [11–13].

As one of the major tasks in academic activities, literature similarity checking is playing an increasingly important role in ensuring the success of scientific research. However, in the mobile computing environment, English literatures as well as their corresponding user-literature reading records should be sent to a remote cloud platform for uniform similarity checking (e.g., through a collaborative filtering manner) in a centralized manner, which raises a heavy burden on the response time of similarity checking in cloud platform since there are so many literatures as well as historical user-literature records in various academic databases. In addition, in the traditional cloud-based literature similarity calculation process, each user’s literature reading records are a kind of sensitive information that calls for certain privacy protection. Therefore, it becomes particularly emergent to seek for other privacy-preserving and lightweight literature similarity checking solutions to accommodate the personalized requirements of researchers in the mobile computing environment. Fortunately, edge computing has shown its unique advantages in processing various big data mining and analysis tasks due to its inherent properties in low data transmission amount and quick response time [14–17]; because in edge computing paradigm, most data are not necessary sent to a cloud center for integration, instead, most data are pre-processed beforehand in the nearby edge servers. This way, the data transmission and response time conditions are improved considerably [18–21].

However, mobile edge computing is still on the early stage after its birth. And therefore, there are still some critical issues that need to be solved well [22–25]. Especially, how to perform lightweight similarity checking of English literatures in the mobile computing environment for easy-to-use mobile office services is a difficult task that needs to be further studied. In view of this challenge, a hash-based index mechanism is introduced in this paper (the time complexity of this hash technique has been proven to be close to O(1)), which is followed by a lightweight literature similarity checking approach based on literature indexes, i.e., $\mathrm {SC_{MEC}}$ is proposed to alleviate the heavy computational costs and transmission costs in mobile academic activities.

Generally, our contributions are three-fold.

(1) An index mechanism is proposed in our paper to aid the literature similarity checking task in the mobile edge computing, in which most literature data from mobile devices are quickly processed by the closer edge servers, without being sent to the remote cloud platform for uniform but time-consuming processed.

(2) An index-based literature similarity checking approach $\mathrm {SC_{MEC}}$ is proposed to support the academic activities on mobile devices. Since the index mechanism is proven to be of a low time complexity, our proposed index-based literature similarity checking approach $\mathrm {SC_{MEC}}$ is very lightweight and time-efficient.

(3) Experiments are deployed for validation and the final experimental results with related approaches prove the effectiveness and efficiency of our $\mathrm {SC_{MEC}}$ in this paper.

Our paper is structured as follows. Related literatures are investigated in Section 2. A three-layer framework is presented in Section 3 to support the literature similarity checking tasks in the mobile edge computing environment. An index-based literature similarity checking approach $\mathrm {SC_{MEC}}$ is put forward in Section 4. Evaluations are presented in Section 5. At last, we summarize the paper in Section 6 which also points out the future research directions.

## Related literature

The literature similarity checking issue in cloud environment has been investigated by researchers. Next, we summarize existing literatures as follows.

In [26], with the advent of cloud computing, data management jobs are outsourced to cloud to save money. However, there are privacy issues. This paper considers the use of cloud to encrypt data before data outsourcing, and conducts similarity search of multiple keywords on outsourced cloud data. The experiment proves that the search design implemented in this paper can effectively resist internal threats and show high performance in cloud search time. In [27], to solve security problem of cloud, the authors bring forth a verifiable privacy-protected multi-keyword text search (MTS) solution based on similarity ranking. Meanwhile, to improve search efficiency of data, this paper also suggested a tree-based index structure as well as a variety of adaptive multidimensional (MD) algorithms, which make the actual search efficiency far better than the linear search efficiency. In [28], aiming at the protection of cloud data privacy, this paper proposed a new similarity based secure data deduplication scheme combining bloom filter and content definition chunking technology. The scheme only deduplicates similar files to significantly reduce computing overhead. In [29], deduplication is widely used in cloud computing to improve space complexity. While the secure mechanisms have some security drawbacks, such as the inability to provide flexible access control. In this paper, the authors propose an encrypted deduplication scheme EDedup based on similarity awareness, which supports flexible revocable access control. EDedup divides the file into segments, which take advantage of similarity to reduce computation overhead through a representative hash algorithm. EDedup also integrates source-based similarity detection and target-based duplicate block detection to protect against attacks and ensure efficient deduplication.

In [30], considering the limitations of classical ICP approaches, this paper proposes a improved iterative nearest point (ICP) approach by the similarity of point cloud curvature features. Based on the classical ICP algorithm, the authors introduce the rough alignment method of principal component analysis, and use k-D tree to segment three-dimensional point cloud to fasten the search process of nearest neighbor points. Experiments show that the approach is more accurate. In [31], more recently, the emergence of new applications using advanced content representation has driven the rise of immersion technology. Among the alternatives available, the point cloud is a promising solution. Here, an effective objective measure is introduced to capture the perceptual degradation of distorted point clouds. The quality assessment of point clouds is mainly based on angular similarity. In [32], the QoS of cloud services will change gradually over time, but the existing service recommendation methods do not pay attention to this problem. Therefore, the authors bring forth a time-aware recommendation method to solve this problem. A new similarity enhanced collaborative filtering method is developed to capture the temporal characteristics of user similarity and solve the problem of data sparsity. Meanwhile, ARIMA model is used for service quality prediction at future time points. In [33], in addition to cloud data storage services, data similarity retrieval is another basic service provided by cloud, especially in image data. This paper proposes a privacy-protecting similar image detection solution based on LSH for image hosted in cloud. This method can effectively secure the sensitive cloud data and obtain better retrieval results.

With the above investigation, a simple conclusion could be drawn that existing literatures are more focusing on the delay, security and privacy of cloud-based literature similarity checking, without considering the migration of literature similarity checking task from cloud to mobile edge, which limits the success of mobile academic activities considerably. Considering this limitation, we put forward an index-based literature similarity checking solution $\mathrm {SC_{MEC}}$ in the next sections.

## Framework

**Fig. 1:**
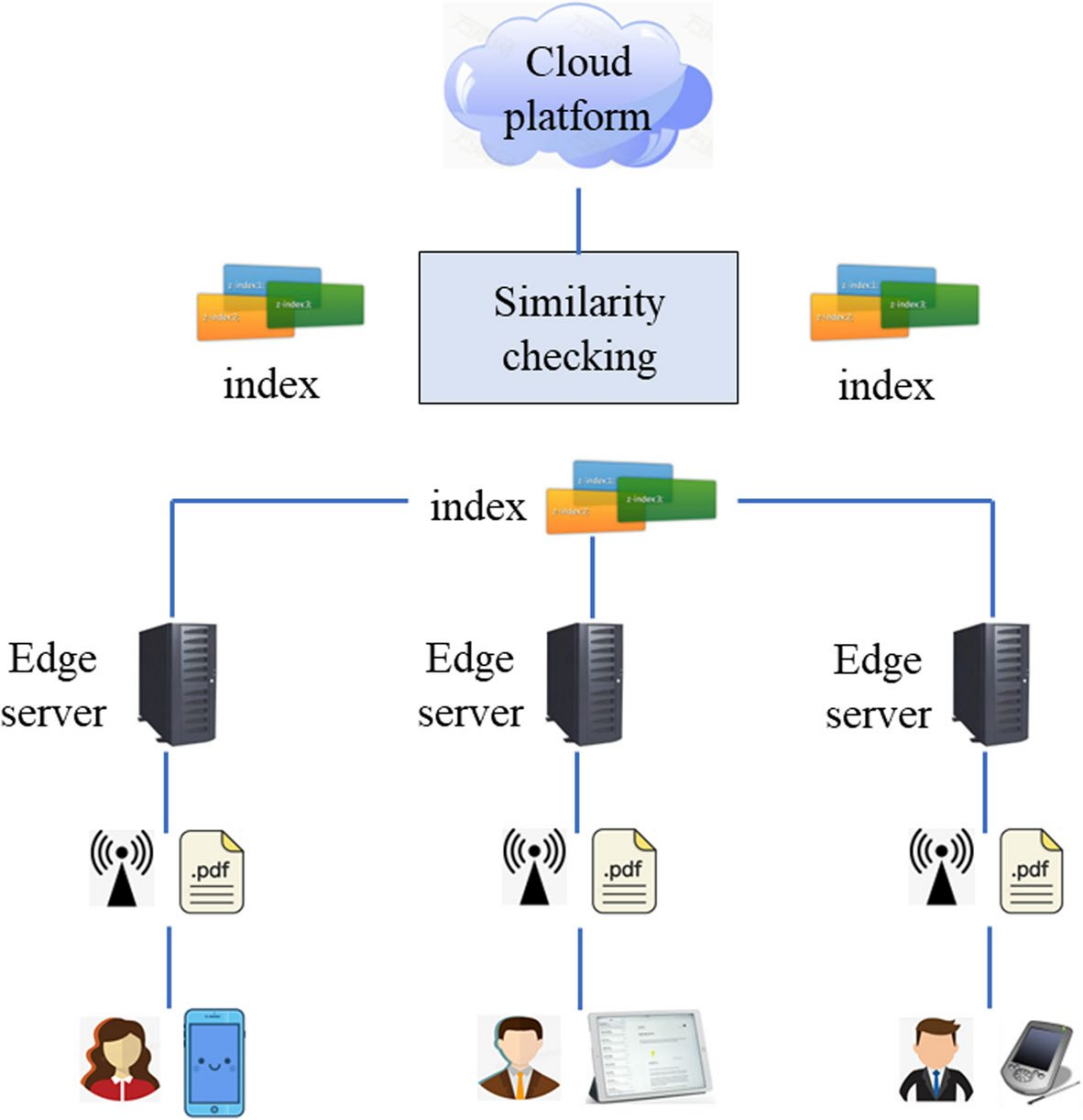
Three-layer framework of literature similarity checking in mobile edge computing

A three-layer framework is presented in Fig. 1 to support the literature similarity checking tasks in the mobile edge computing environment. As Fig. 1 shows, researchers use various mobile devices (e.g., mobile phone, iPAD, PDA and so on [34–36]) for different academic activities. During this process, the academic literatures on mobile devices [37, 38] (e.g., PDFs) are not necessary to be transmitted directly to the remote cloud platform. Instead, researchers transmit their academic literatures to nearby edge servers through close mobile devices via wireless communication technology. Afterwards, each edge server is responsible for converting the academic literatures hosted in the edge server into a corresponding index (we call it literature index here). The literature index here is a lightweight embedding of the literature compared to the literature itself. Each edge server records its converted literature indexes and transmits them to a cloud platform that is responsible for processing all the literature indexes transmitted from all edge servers. Finally, the cloud platform checks the literature similarity via comparing the literature indexes which are very short and lightweight. This way, we can achieve the literature similarity checking task through the three-layer device-edge-cloud framework presented in Fig. 1.

## Index-based similarity checking in mobile edge computing: $\mathrm {SC_{MEC}}$

According to the three-layer framework of literature similarity checking task presented in Fig. 1, our proposed literature similarity checking approach $\mathrm {SC_{MEC}}$ can be divided into two steps: first, we need to convert each literature from mobile devices into its corresponding index; second, we need to send the literature indexes to the central cloud platform for uniform similarity checking. Next, we introduce the major two steps of $\mathrm {SC_{MEC}}$ approach.

**Step 1: Conversion from literatures to indexes.**

Traditional literature similarity checking approaches need to make comparisons between different literatures in a direct and straightforward way, e.g., word by word, sentence by sentence, paragraph by paragraph, etc. Such a direct and straightforward literature comparison way is often time-consuming and tiresome [39, 40], which probably decreases the satisfaction degree of researchers who often expect a quick and accurate literature similarity checking result. Therefore, to speed up the above literature similarity checking process, we need to convert the initial literatures which are often long into corresponding shorter embeddings. Since the content of each literature is often much, we convert each literature into a short index or embedding. This way, we can evaluate whether two literatures are similar or not by comparing their index values instead of their literature contents. The advantage of such an operation is that we can minimize the time cost for similar literature evaluation and discovery. Here, we adopt the classic Simhash and LSH techniques (the time complexity of the mentioned Simhash and LSH techniques have been proven to be close to O(1)). In our proposal, we use Simhash technique to convert each literature into a corresponding Boolean vector, whose purpose is to convert the text information of each literature into a corresponding 0/1 string that is easy to process and calculate in the subsequent similar literature evaluation process.

Next, we introduce the concrete step of conversion from literatures to indexes. First, we use Simhash technique to convert each literature (here, the literature set is denoted by $Lit_{Set}$ = $(lit_1, \dots , lit_n)$) into a long signature (here, the signature set for the literatures in $Lit_{Set}$ is denoted by $Sig_{Set}$ = $(sig_1, \dots , sig_n)$). The concrete conversion process is introduced in detail at follows.

**(1) Word segmentation for literatures.**

Each literature often includes many words, which makes it hard to calculate the similarity degree between different literatures directly. To tackle this issue, we first convert a long literature into a word vector through various mature word segmentation tools in natural language processing (NLP) domain, e.g., word2vec or fastText. The concrete word segmentation process will not be introduced in detail here. Interested readers can refer to the related literatures in NLP. Here, we take a literature *lit* as an example for illustration. We assume that the literature *lit* is converted into a word vector $V_{lit}$ as specified in (1) based on word2vec or fastText. Here, *m* is not a fixed value since different literatures often include different word number after word segmentation.

1 $$\begin{aligned} V_{lit} = (a_1, \dots , a_m) \end{aligned}$$

**(2) Hash projection from a word vector to a 0/1 vector.**

In the last substep, we have converted each literature *lit* into a word vector $V_{lit}$. However, it is challenging to evaluate the similarity between word vectors. To tackle this issue, we further convert the word vector $V_{lit}$ of literature *lit* into a numerical vector $NumV_{lit}$. The concrete conversion process is based on any hash projection table. Here, for simplicity, we use the classic ASCII coding table adopted widely in computer domain to achieve the goal of hash projection. For example, if $V_{lit} = (a_1, a_2)$, $a_1$ = 11110000 and $a_2$ = 10101010, then $NumV_{lit}$ = (1 1 1 1 0 0 0 0 1 0 1 0 1 0 1 0). Next, we replace the “0” entries in the vector by “-1” and then derive a new vector constituted by “1” and “-1” entries only. For example, $NumV_{lit}$ = (1 1 1 1 0 0 0 0 1 0 1 0 1 0 1 0) is updated to be $NumV_{lit}$ = (1 1 1 1 -1 -1 -1 -1 1 -1 1 -1 1 -1 1 -1).

**(3) Vector weighting.**

Weight significance is often inevitable in many applications involving multiple dimensions or criteria [41–43]. Inspired by the above analysis, in a literature, each word should be assigned a concrete weight value indicating the importance and significance of the word in depicting the whole literature. The weights of words in a literature could be generated in many ways such as TF/IDF, which is not repeated here. Here, we assume that weight vector corresponding to the *m* words in vector $V_{lit}$ in (1) is *W* as specified in (2)-(3).

2 $$\begin{aligned} W_{lit} = (w_1, \dots , w_m) \end{aligned}$$

3 $$\begin{aligned} \sum \limits _{j = 1}^m {{w_j}} = 1 \end{aligned}$$

**(4) Vector union by addition.**

For a literature *lit*, with its word vector $V_{lit}$ = $(a_1, \dots , a_m)$ in Eq. (1) (here, please note that the hash projection from 0 to -1 has been applied to the word vector) and its weight vector $W_{lit} = (w_1, \dots , w_m)$ in Eq. (2), we can make a dot production operation between vectors $V_{lit}$ and $W_{lit}$, whose result is denoted by $DP_{lit}$ in (4). For example, if $V_{lit}$ = $(a_1, a_2)$, $a_1$ = 11110000, $a_2$ = 10101010, $w_1$ = 0.4, $w_2$ = 0.6, then $DP_{lit}$ = (1 1 1 1 -1 -1 -1 -1) * 0.4 + (1 -1 1 -1 1 -1 1 -1) * 0.6 = (0.4 0.4 0.4 0.4 -0.4 -0.4 -0.4 -0.4) + (0.6 -0.6 0.6 -0.6 0.6 -0.6 0.6 -0.6) = (1 -0.2 1 -0.2 1 -0.2 1 -0.2).

4 $$\begin{aligned} DP_{lit} = (b_1, \dots , b_m) = V_{lit} * W_{lit} = \sum \limits _{j = 1}^m {{a_j}*{w_j}} \end{aligned}$$

**(5) Dimension reduction.**

In the above substep, we have converted each literature *lit* into a corresponding $DP_{lit} = (b_1, \dots , b_m)$ by Eq. (4). However, from the dimension perspective, each entry in vector $DP_{lit}$ can take any real value and therefore, its value range is often very large and not suitable for subsequent similarity calculation and evaluation. To overcome this shortcoming, we reduce each dimension’s value range as follows, since binary embedding is widely applied to various big data scenarios to reduce the search and processing time [44–46]. In concrete, we make the following conversions in Eq. (5). Afterwards, each entry $b_j$ in vector $DP_{lit}$ is equal to either 1 or 0, which narrows the value range of vector $DP_{lit}$ significantly. This way, we successfully achieve the goal of dimension reduction.

5 $$\begin{aligned} b_j = \left\{ \begin{array}{rl} 1 &{} \text {if } b_j > 0,\\ 0 &{} \text {if } b_j \le 0. \end{array} \right. ( j = 1, 2,\dots , m ) \end{aligned}$$

**(6) Deep dimension reduction by LSH.**

To further reduce the dimensions of $DP_{lit}$ for each literature *lit*, we use LSH technique to build a deep index for each *lit*. Concretely, we generate an *m*-dimensional vector $X = (x_1, \dots , x_m)$ randomly by Eq. (6), where each entry $x_j$ of vector *X* belongs to range [-1, 1]. Next, we convert the *m*-dimensional vector $DP_{lit}$ into a smaller *r*-dimensional vector$Z (r \ll m)$ by Eqs. (7)-(9). Here, the purpose of Eq. (7) is to calculate the projection from the original vector $DP_{lit}$ to the vector *X*; the purpose of Eq. (8) is to reduce the dimensions involved; afterwards, we repeat the operations in Eqs. (7) and (8) *r* times to obtain $z_1, \dots , z_r$. Then we can get a new vector $Z = (z_1, \dots , z_r)$ which is much shorter than the original vector $DP_{lit}$. This way, we successfully achieve the goal of dimension reduction. In addition, the LSH technique has been proven a lightweight nearest neighbor discovery approach whose time complexity is approximately O(1). Therefore, the proposed LSH-based similar literature discovery approach is very suitable for the big data context.

6 $$\begin{aligned} x_j = random (-1, 1) \end{aligned}$$

7 $$\begin{aligned} z = DP_{lit}*X =\sum \limits _{j = 1}^m {{b_j}*{x_j}} \end{aligned}$$

8 $$\begin{aligned} z = \left\{ \begin{array}{rl} 1 &{} \text {if } z > 0,\\ 0 &{} \text {if } z \le 0. \end{array} \right. \end{aligned}$$

9 $$\begin{aligned} Z = (z_1, \dots , z_r) \end{aligned}$$

**Step 2: Similarity checking of literatures based on indexes.**

In Step 1, for each literature in $Lit_{Set}$ = $(lit_1, \dots , lit_n)$, we have obtained a corresponding hash index $Z_j ( j =1, 2, \dots , n )$. Next, we compare any two literatures $lit_i$ and $lit_j$ $( 1 \le i \le n, 1\le j \le n)$ through comparing their respective hash indexes $Z_i$ and $Z_j$. In concrete, if $Z_i = Z_j$ holds, we can simply conclude that literatures $lit_i$ and $lit_j$ are similar with high probability. However, the above literature similarity evaluation solution is not always correct since LSH is a probability-based neighbor search technique. To minimize the negative influences incurred by probability, for each literature $lit_j$ in $Lit_{Set}$, we do not generate only one hash index $Z_j$; instead, we generate *h* indexes $Z_{j}^1, \dots , Z_{j}^h$. Afterwards, the similarity between literatures $lit_i$ and $lit_j$ is evaluated by Eq. (10). This way, we can evaluate whether two literatures are similar or same based on their respective hash indexes, to improve the literature similarity evaluation efficiency.

10 $$\begin{aligned} lit_i \,\, \text {and} \,\, lit_j \,\, \text {are similar iff} \,\, \exists \,\, k \,\, \text {satisfying} \,\, Z_{i}^k = Z_{j}^k (1 \le k \le h) \end{aligned}$$

The details of our proposed $\mathrm {SC_{MEC}}$ algorithm can be described clearly by the following pseudo code.

**Figure:**
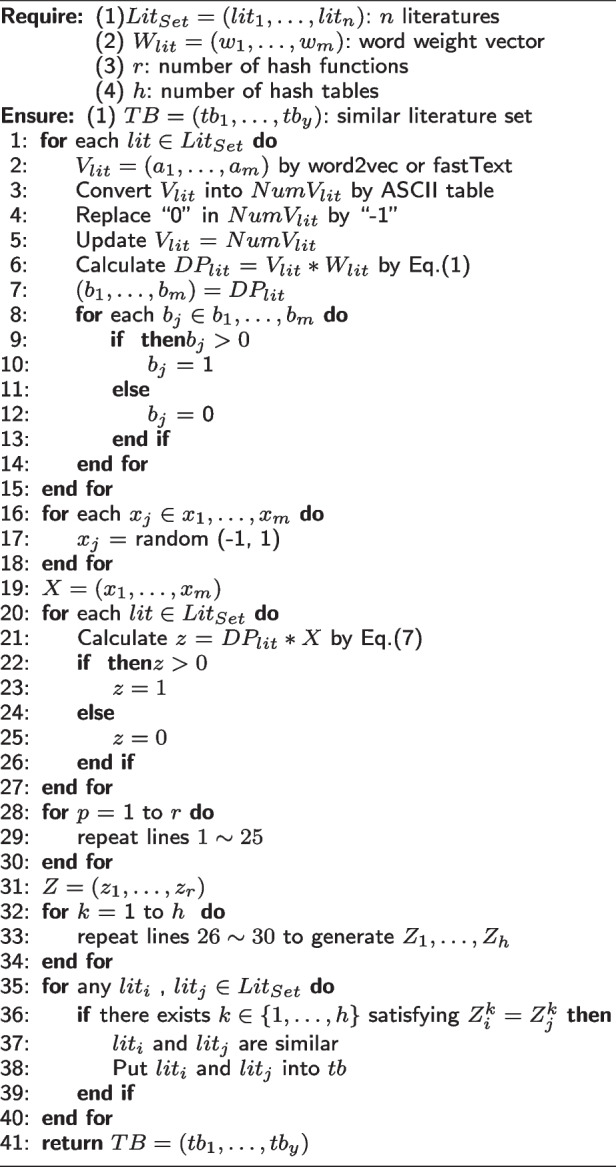
**Algorithm 1** $$\mathrm {SC_{MEC}}$$

## Evaluation

Next, we prove the feasibility of $\mathrm {SC_{MEC}}$ algorithm in handling literature similarity checking in mobile edge computing environment. Concretely, the experiments are deployed on WS-DREAM dataset^1^ which records a group of services as well as their quality performances. Here, we use the records in the dataset to simulate the literatures as well as their digital signatures. Two classic similar neighbor searching methods are used for comparison purpose, i.e., UCF and ICF. Experiment hardware includes a 2.50 GHz processor and 16.0 GB memory; experiments are executed under WIN-7 operation system and Python-3. We run each experiment 100 times and record their average performance for result display. Please note that the parameter settings in this paper are determined in an experienced way, i.e., by experiment tests.

**Test 1: Computational time comparison.**

**Fig. 2:**
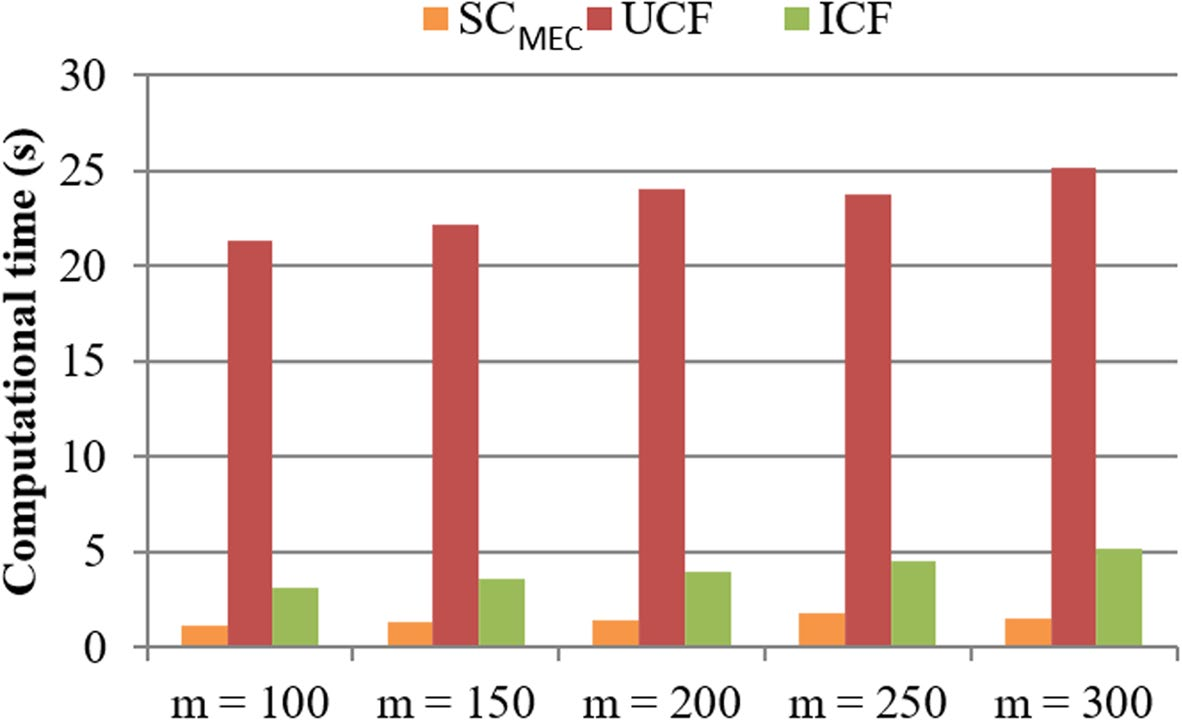
Computational time comparison (w.r.t. *m*)

**Fig. 3:**
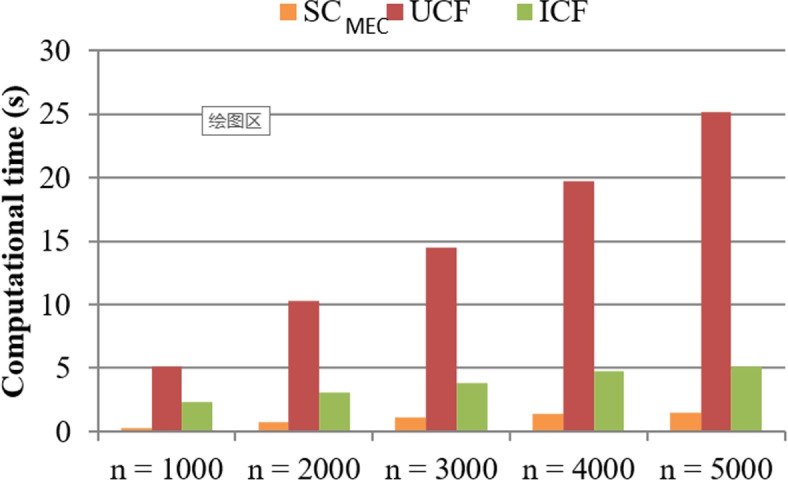
Computational time comparison (w.r.t. *n*)

In the big data context, data processing speed is very important since response time is an influencing factor associated with user experience [47, 48]. Inspired by this observation, we test the speed of $\mathrm {SC_{MEC}}$ with baseline methods: UCF and ICF. Test results are presented in Figs. 2 and 3, respectively. In concrete, in Fig. 2, time cost of three methods is compared with the size of signature dimensions, i.e., m. Here, *m* varies from 100 to 300; *n* is equal to 5000; number of functions in $\mathrm {SC_{MEC}}$, i.e., *r* = 10; number of tables in $\mathrm {SC_{MEC}}$, i.e., *k* = 18. In Fig. 2, the time costs of three methods approximate rise with the rising of m since more calculation operations are necessary in all three methods when there are more signature dimensions of literatures. Moreover, compared to UCF and ICF methods, our proposed $\mathrm {SC_{MEC}}$ algorithm consumes less time. This is due to the fact that UCF and ICF need to calculate literature similarity based on collaborative filtering idea whose time complexity is relatively high; while the $\mathrm {SC_{MEC}}$ algorithm first generates literature indexes offline and then uses existing literature indexes to discover similar literatures. Therefore, $\mathrm {SC_{MEC}}$ often performs better than UCF and ICF in terms of consumed time. Similar comparison results could be observed in Fig. 3 where the computational time of three methods is compared with the number of literatures, i.e., n. Here, *n* varies from 1000 to 5000, *m* is equal to 300, *r* = 10, *k* = 18. The reason is not repeated again.

**Fig. 4:**
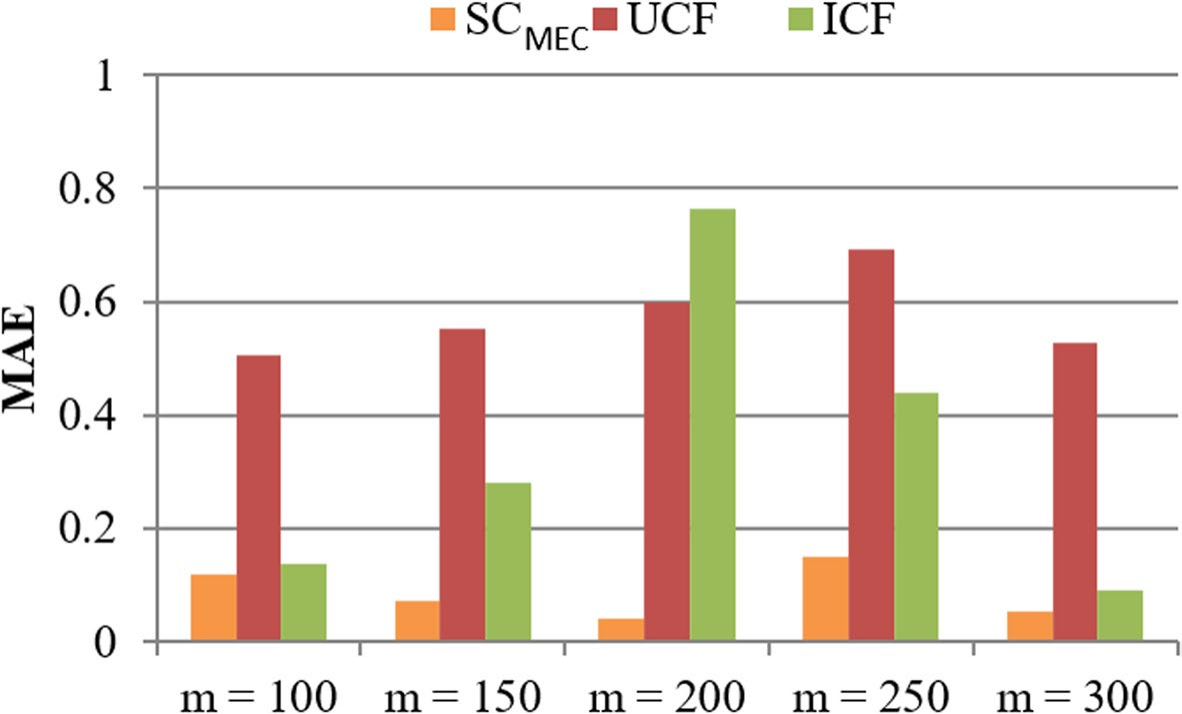
Accuracy comparison (w.r.t. *m*)

**Fig. 5:**
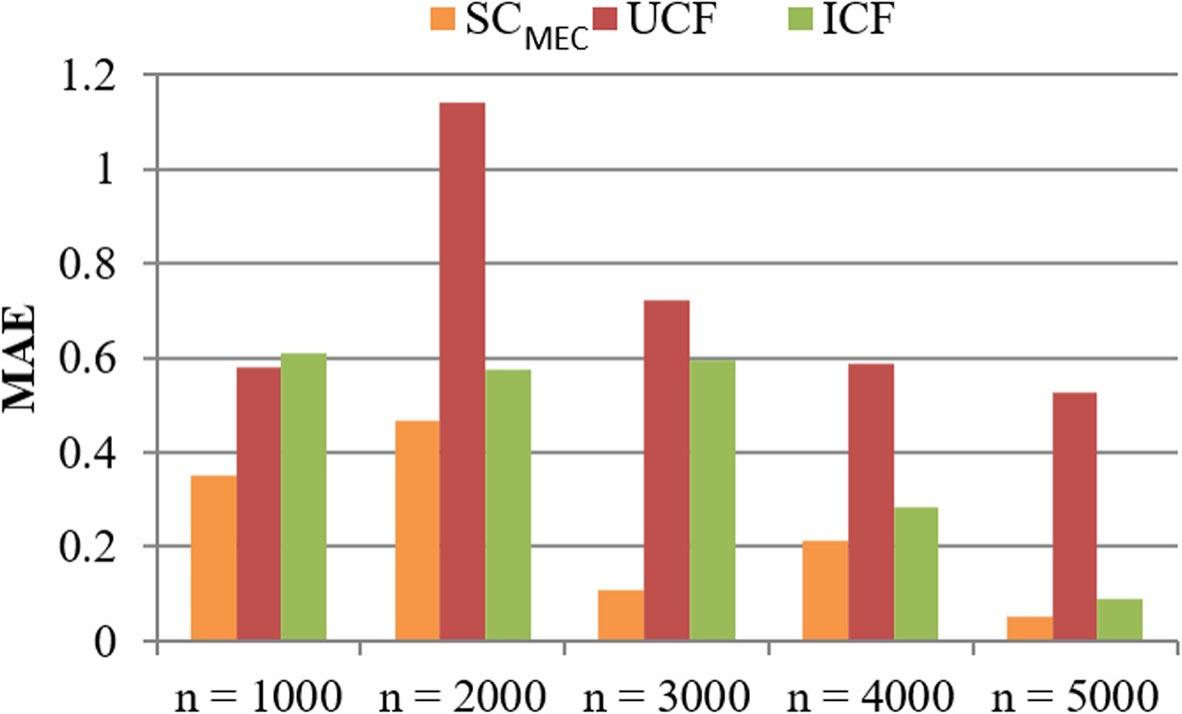
Accuracy comparison (w.r.t. *n*)

**Test 2: Accuracy comparison.** Here, we measure the literature similarity checking accuracy (in the form of MAE, smaller is better) of three methods, whose results are presented in Figs. 4 and 5, respectively. In concrete, in Fig. 4, *m* = $\{100, \dots , 300\}$, *n* = 5000, *r* = 10, *k* = 18. The results reported in Fig. 4 shows that the accuracy of three methods all varies with the growth of *m* and $\mathrm {SC_{MEC}}$ algorithm outperforms UCF and ICF methods in accuracy. The reason is that in $\mathrm {SC_{MEC}}$, the adopted hash indexes of literatures can guarantee to discover all the most similar literatures. As a result, the accuracy of $\mathrm {SC_{MEC}}$ is the highest and the MAE of $\mathrm {SC_{MEC}}$ is the lowest. Such a comparison result indicates a good literature similarity checking performance of the proposed $\mathrm {SC_{MEC}}$ method. Similar results could also be observed in Fig. 5 where $n = \{1000, \dots , 5000\}$, *m* = 300, *r* = 10, *k* = 18. Figure 5 shows that the accuracy of three methods approximately rises when *n* (i.e., MAE of three methods declines when *n* rises) grows. The reason can be analyzed as follows: when *n* becomes larger, there are more literatures as well as their associated signatures; in this situation, more valuable information taking part in literature similarity checking is available and hence, the accuracy is enhanced accordingly. Furthermore, the MAE of $\mathrm {SC_{MEC}}$ is generally lower than UCF and ICF, which indicates a better performance of $\mathrm {SC_{MEC}}$ in literature similarity checking.

**Test 3: Performances of** $\mathrm {SC_{MEC}}$.

**Fig. 6:**
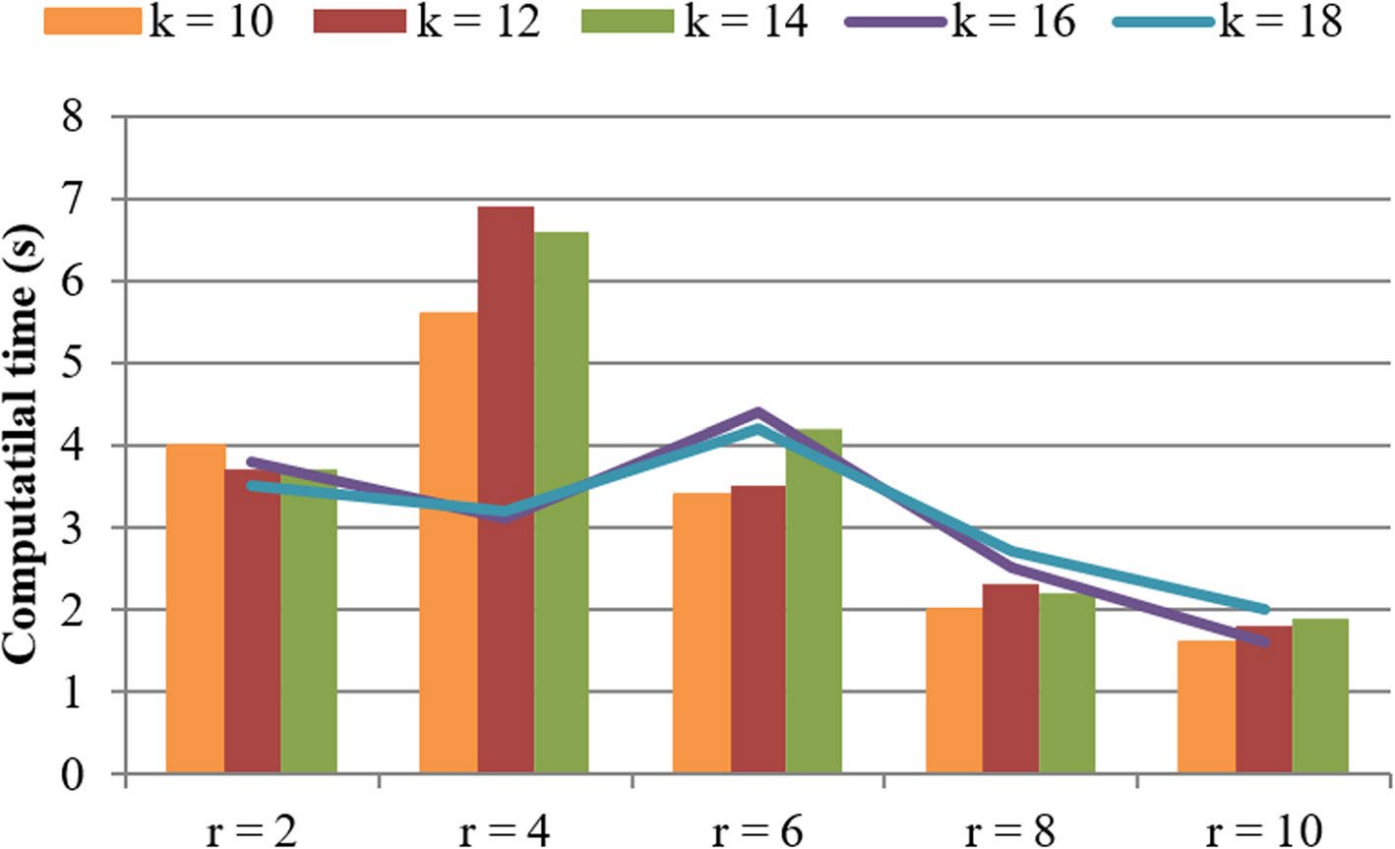
Computational time of our algorithm (w.r.t. *r* and *k*)

**Fig. 7:**
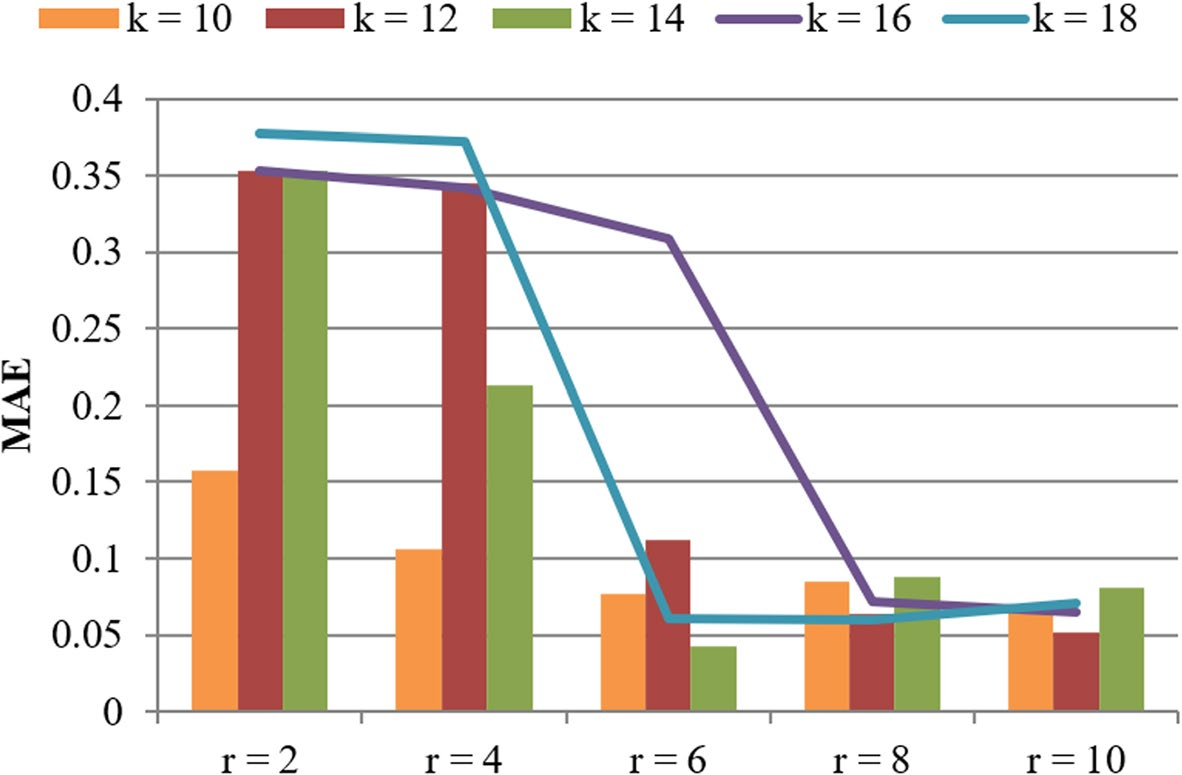
Accuracy of our algorithm (w.r.t. *r* and *k*)

As introduced in Section 4, there are several key influencing factors that can determine the performances of $\mathrm {SC_{MEC}}$, such as parameters *r* and *k*. To observe the relationships between the influencing factors and the algorithm performances, several experiments are designed where *n* and *m* are equal to 5000 and 300, respectively; *r* varies from 2 to 10 and *k* varies from 10 to 18. Concrete data are reported in Figs. 6 and 7. In concrete, Fig. 6 shows the computational time of $\mathrm {SC_{MEC}}$ when the parameters *r* and *k* fluctuate, where the computational time approximately drops with the increment of *r* and the consumed time approximately drops with the increment of parameter *k*. Next, we discuss the reason behind such a conclusion. First, if we use more hash functions (i.e., larger *r*) used to calculate literature similarity, the more probable that the top-similar literatures be discovered; in this situation, the finally returned similar literatures are usually fewer and hence, less time is consumed accordingly. Second, if we use more hash tables (i.e., larger *k*) used to calculate literature similarity, more similar literatures are probably discovered; in this situation, the finally returned similar literatures are usually more and hence, more time is consumed accordingly.

Figure 7 shows the accuracy of $\mathrm {SC_{MEC}}$ when the parameters *r* and *k* fluctuate, where the accuracy value approximately drops with the increment of parameter *r* and the accuracy varies with the increment of parameter *k*. Next, like in Fig. 6, we discuss the reason behind such an observation from Fig. 7. If we use more hash functions (i.e., larger *r*) to calculate literature similarity, the returned similar literatures are “more similar”; accordingly, literature similarity checking accuracy is higher (i.e., MAE is lower).

## Conslusions

With the advent of information age, mobile devices have become one of the major convenient equipment that aids people’s daily office activities such as academic research, one of whose major tasks is to check the repetition rate or similarity among different English literatures. Traditional literature similarity checking solutions in cloud paradigm often call for intensive computational cost and long waiting time. To tackle this issue, in this paper, we modify the traditional literature similarity checking solution in cloud paradigm to make it more suitable for the light-weight mobile edge environment. Furthermore, we put forward a lightweight similarity checking approach $\mathrm {SC_{MEC}}$ for English literatures in mobile edge computing environment. To validate the advantages of $\mathrm {SC_{MEC}}$, we have designed massive experiments on a dataset. The reported experimental results show that $\mathrm {SC_{MEC}}$ can deliver a satisfactory similarity checking result of literatures compared to other existing approaches.

Literature similarity checking in mobile edge computing environment often involves certain sensitive user privacy [49]. Therefore, in future study, we will further consider the privacy disclosure issue in our proposed algorithm by introducing more effective privacy protection techniques including blockchain, federated learning, DP, encryption, etc. [50–52]. In addition, energy saving and cost optimization are also key challenges in typical big data applications [53, 54]. Hence, we will introduce more classic performance optimization and computational offloading technologies.

## Data Availability

http://wsdream.github.io/

## Abbreviations

MTS: Multi-keyword text search
ICP: Iterative nearest point
MD: Adaptive multidimensionaL
LSH: Locality-Sensitive Hashing
NLP: Natural language processing
MEC: Mobile edge computing

## Footnotes

1 https://wsdream.github.io/
